# Risk prediction models for sepsis-associated encephalopathy: a systematic evaluation and meta-analysis

**DOI:** 10.7717/peerj.20770

**Published:** 2026-02-13

**Authors:** Ting ting He, Tuo quan Jiao, Xue mei An

**Affiliations:** 1 School of Nursing, Chengdu University of Traditional Chinese Medicine, Chengdu, China; 2 Hospital of Chengdu University of Traditional Chinese Medicine, Chengdu, China; 3 Deyang Hospital Affiliated to Chengdu University of Traditional Chinese Medicine, Deyang, China

**Keywords:** Sepsis-Associated Encephalopathy, Meta analysis, Systematic review, Risk, Prediction model

## Abstract

**Background:**

The number of risk prediction models for sepsis-associated encephalopathy (SAE) is increasing, while the quality and applicability of these models in clinical practice and future research remain uncertain.

**Objective:**

To systematically review published studies on SAE risk prediction models.

**Design:**

Systematic review and meta-analysis of observational studies.

**Methods:**

A systematic search of PubMed, Web of Science, Embase, Wanfang, VIP, and CNKI databases was conducted from inception to April 2, 2025, to identify studies on SAE risk prediction models. Two independent reviewers screened the studies and extracted data. The Prediction model Risk Of Bias Assessment Tool (PROBAST) was applied to evaluate the risk of bias and applicability of the included studies.

**Results:**

A total of 1,994 studies were identified, and 10 were included after screening. The reported incidence of SAE ranged from 15.16% to 63.3%. Age and Sequential Organ Failure Assessment (SOFA) score are the most frequently adopted factors with significant predictive value, both of which were incorporated into five models. Both the SOFA score and age were significantly associated with SAE. In studies with available data, the odds ratio (OR) for age ranged from 1.084 to 1.018, while that for SOFA score ranged from 1.246 to 2.416. The area under the receiver operating characteristic curve (AUC) for the 10 studies ranged from 0.743 to 0.975. All studies were found to have a high risk of bias, primarily due to inappropriate data sources and deficiencies in the analysis domain. The pooled AUC for the six validated models was 0.83 (95% confidence interval [0.77–0.89]), indicating fair discrimination.

**Conclusion:**

Although the included studies reported some discrimination in the SAE prediction models, all were found to have a high risk of bias according to the PROBAST checklist.

**Registration:**

This study protocol was registered on PROSPERO (registration number: CRD420251012485).

## Introduction

Sepsis, defined as life-threatening organ dysfunction caused by a dysregulated host response to infection, remains one of the leading causes of mortality in the intensive care unit (ICU) and continues to pose a substantial global health burden, particularly in low- and middle-income countries (Aljefri et al., 2023; Hotchkiss & Karl, 2003). Sepsis-associated encephalopathy (SAE), a diffuse brain dysfunction occurring in the absence of direct central nervous system infection, affects approximately 8% to 70% of patients with sepsis and is associated with 28-day mortality rates of up to 46% (Lei & Wu, 2025; Li et al., 2025; Mazeraud et al., 2020).

The absence of standardised diagnostic criteria, combined with confounding factors such as sedation and mechanical ventilation, poses major challenges for recognising SAE (Dumbuya et al., 2023; Mazeraud et al., 2020; Shirodkar et al., 2025). Its non-specific symptoms (*e.g.*, delirium, coma) can be misattributed to other encephalopathies, risking misdiagnosis. Delayed or missed diagnosis often contributes to higher mortality and an increased risk of neurological complications. Therefore, early identification of patients at risk for SAE is crucial, as it enables timely interventions to mitigate long-term complications.

Despite the growing number of SAE prediction models, their methodological quality and clinical utility remain uncertain. This study systematically reviews and meta-analyses SAE risk prediction models to evaluate their risk of bias, performance, and applicability, providing an evidence-based foundation for future model development and clinical implementation.

### Methods

The study protocol was registered on PROSPERO (registration number: CRD420251012485). Literature screening was conducted on April 2, 2025, and data extraction was conducted on April 10, 2025.

### Search strategy

The literature search was conducted in accordance with the Preferred Reporting Items for Systematic Reviews and Meta-Analyses (PRISMA) 2020 guidelines and was independently verified by two reviewers (TT and TQ) to ensure the comprehensiveness and reproducibility of the search results (see Table S1 for details). The PubMed, Web of Science, Embase, Wanfang, VIP, and CNKI databases were searched for studies on SAE risk prediction models, with a search timeframe from inception to April 2, 2025, for all databases. The search terms included “sepsis-associated encephalopathy, sepsis-associated psychosis, sepsis encephalopathy prediction, early warning, predictor, influencer, influencing factor, risk assessment, risk prediction, modeling, tool, column-line graph, nomogram”. We also identified other relevant studies through the reference lists of retrieved studies and review articles.

For systematic evaluation, we adopted the PICOTS framework, and the key items of our systematic review are described below:

P (Population): Adult patients (≥18 years old) with sepsis, as defined by the Sepsis 3.0 international consensus criteria (Singer et al., 2016). The setting included Intensive Care Unit (ICU), emergency departments, and general wards. Patients with other primary causes of encephalopathy were excluded from the study.

I (Intervention model): Risk prediction model for SAE.

C (Comparator): No competing model.

O (Outcome): Occurrence of SAE, rather than subgroup outcomes such as sepsis-associated delirium.

T (Timing): Assessed within 24 h of hospital admission.

S (Setting): Intended use was to predict SAE.

### Inclusion and exclusion criteria

Inclusion criteria: (1) Study subjects: Adult patients (≥18 years old) diagnosed with sepsis according to the Sepsis 3.0 consensus criteria were included. Specifically, studies were eligible if sepsis was defined as a suspected or documented infection accompanied by an increase in the Sequential Organ Failure Assessment (SOFA) score of ≥2 points (Singer et al., 2016); (2) Study types: cohort studies, case-control studies and cross-sectional studies; (3) Study content: development of a predictive model for the risk of SAE; (4) Outcome indicators: the occurrence of SAE. Exclusion criteria: (1) Animal or cell-based experiments, reviews, and conference papers; (2) Studies that only analyse the predictive capability of influencing factors without constructing SAE prediction models; (3) Not written in English or Chinese.

Given the absence of a universally accepted diagnostic gold standard, studies were included if SAE was defined as brain dysfunction occurring in the context of sepsis, after excluding other causes such as metabolic, structural, or drug-induced encephalopathy. In most included studies, SAE diagnosis was based on a Glasgow Coma Scale (GCS) score <15 and/or the presence of delirium as assessed by the Confusion Assessment Method for the ICU (CAM-ICU).

### Literature screening and data extraction

To ensure the study’s objectivity, two reviewers (TT and TQ) independently screened the literature and extracted the data. In case of disagreement, a third party (XM) was asked to discuss or consult to resolve the issue. EndNote was used to manage retrieved citations. Data extraction followed the Critical Appraisal and Data Extraction for Systematic Reviews of Prediction Modelling Studies (CHARMS) checklist (Moons et al., 2014). Extracted items included:

(1) Basic information: including authors, year of publication, study design, study population, data source and sample size.
(2) Model information: including handling of missing data, variable selection methods, model development methods, model validation methods, model performance measures, final predictor variables, and model presentation.

### Quality evaluation of models

Two independent researchers (TT and TQ) assessed the risk of bias, applicability, and overall quality of the included studies using the Prediction model Risk Of Bias Assessment Tool (PROBAST) (Moons et al., 2019) and the Grading of Recommendations, Assessment, Development, and Evaluation (GRADE) system (Guyatt et al., 2008). In the event of disagreement, a third party (XM) was asked to resolve the issue through discussion or consultation. PROBAST evaluates four domains—participants, predictors, outcomes, and analysis—each rated as low, high, or unclear risk of bias; applicability concerns were judged across the first three domains. GRADE classifies the certainty of evidence as high, moderate, low, or very low, taking into account study design, risk of bias, inconsistency, indirectness, imprecision, and publication bias.

### Data synthesis and statistical analysis

We performed a meta-analysis of AUC values from validated models using R. To assess heterogeneity among the included studies, we used the *I*^2^ statistic. If *I*^2^ < 50% and *p* > 0.10, it means that the heterogeneity among studies is not significant, and the fixed-effects model was used for the merger; if *I*^2^ ≥ 50% or *p* ≤ 0.1, it means that the heterogeneity among studies is significant, in which case sensitivity analysis will be performed (Lehrer et al., 2023).

After excluding the literature article by article, if heterogeneity persisted, the random-effects model was used for meta-analysis. If the merged effect value did not change significantly, the results of the meta-analysis were deemed stable. Egger’s test (Egger et al., 1997) was used to identify publication bias. *p* > 0.05 indicated a low probability of publication bias.

## Results

### Literature screening process and results

**Figure 1:**
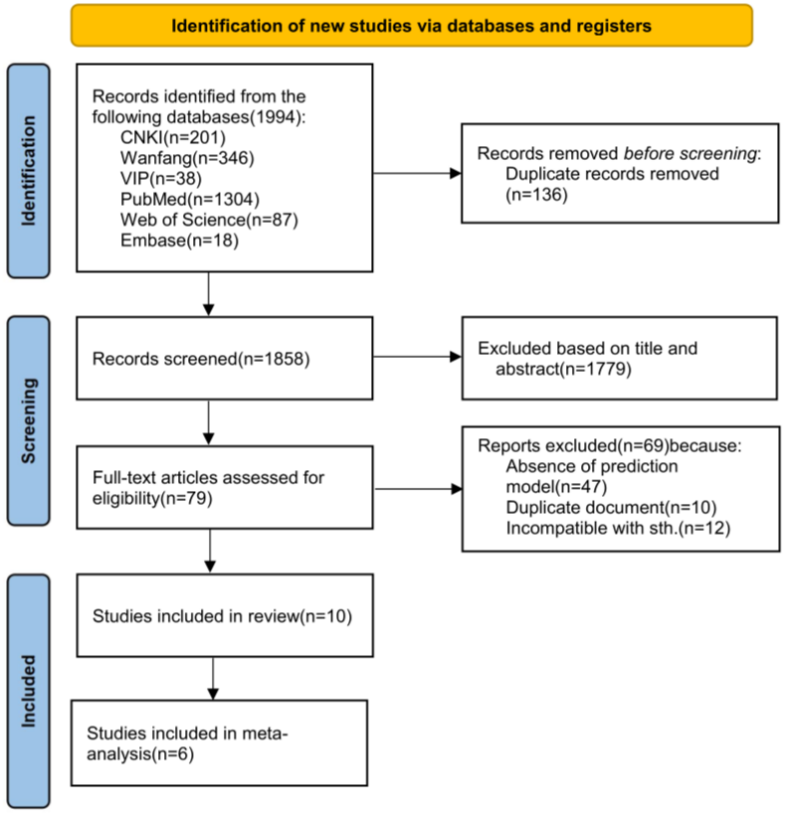
PRISMA flowchart of the study screening process.

A total of 1,994 records were retrieved from all databases. After rigorous screening and eligibility assessment, 10 studies met the inclusion criteria, of which six provided model data suitable for the meta-analysis (Fig. 1).

### Characteristics of included studies

Table 1 summarises the design and subject characteristics of the 10 included studies. The 10 included studies, published between 2021 and 2024, comprised five based on Chinese cohorts and five using U.S. databases. Eight studies were retrospective, and only two were prospective. Regarding the study population, nine studies enrolled adult patients with sepsis, while one focused on elderly patients (≥65 years). Among the ten included studies, the sample size ranged from 67 to 22,361 participants (median: 1,587.5; IQR: 130–893.7). The reported incidence of SAE across these studies ranged from 15.16% to 63.3% (median: 44.7%).

**Table 1:** Overview of basic data of the included studies.

Author (year)	Country of data source	Study design	Participants	Data source	Main outcome	SAE cases/ sample size (%)
Wang, Ziwen^a^ (2023)	China	Retrospective cohort study	Patients with sepsis admitted to the ICU for the first time	ICU of a hospital	SAE	97/640 (15.16%)
Zhou, Hangxiang^a^ (2023)	China	Retrospective cohort study	Patients with sepsis > 18 years of age	ICU of a hospital	SAE	84/213 (39.44%)
Zhao, Qing (2023)	United States	Retrospective cohort study	ICU sepsis patients ≥ 65 years of age	MIMIC-IV	SAE	8,290/22,361 (37.1%)
Xiao, Lu (2022)	United States	Retrospective cohort study	Patients with sepsis ≥ 18 years of age	MIMIC-IV	SAE	4,684/8,935 (52.4%)
Zhang, Li^a^ (2024)	China	Retrospective cohort study	Sepsis patients	ICU of a hospital	SAE	52/130 (40.00%)
Jin, Jun (2024)	United States	Retrospective cohort study	Patients with sepsis ≥ 18 years of age	MIMIC-IV	SAE	2781/4476 (62.1%)
Zhao, Lina (2021)	United States	Retrospective cohort study	Patients with sepsis ≥ 18 years of age	MIMIC III	SAE	1,055/2,535 (41.6%)
Liu, Xiaoyu^a^ (2021)	China	Prospective cohort study	Patients with sepsis ≥ 18 years of age	ICU and emergency department at a hospital	SAE	57/90 (63.3%)
Mei, Jiangjun (2024)	China	Prospective cohort study	Patients with sepsis ≥ 18 years of age	ICU of a hospital	SAE	32/64 (47.8%)
Ge, Chenglong (2022)	United States	Retrospective cohort study	Patients with sepsis ≥ 18 years of age	MIMIC III	SAE	6,284/12,460 (50.4%)

**Notes.**

a Study was published in Chinese.

Note. Wang, Zhao & Chao, 2023; Zhao et al., 2023; Zhou et al., 2023; Lu et al., 2022; Zhang et al., 2024; Jin et al., 2024; Zhao et al., 2021; Liu, 2021; Mei et al., 2024; Ge et al., 2022.

Table 2 presents details of the included prediction models. Most studies (*n* = 9) constructed multivariable logistic regression models, while two applied machine-learning (ML) algorithms. The most frequently used predictors included age (5 models), SOFA score (5 models), serum sodium (Na^+^) (4 models), and body temperature, SpO_2_, Acute Physiology and Chronic Health Evaluation II (APACHE II) score, and serum albumin (each in 3 models).

**Table 2:** Overview of the information of the included prediction models.

Author (year)	Missing data handing	Variable selection	Model development method	Calibration method	Validation method	Final predictors	Model performance	Model presentation
Wang, Ziwen (2023)	–	Forward LR	Logistic regression model	Hosmer– Lemeshow test	Internal validation	Age, Use Boosters, Albumin, SpO2, S100β	A,0.810 (0.763–0.857) B,0.813 (0.740–0.885)	Nomogram model
Zhou, Hang xiang (2023)	–	Stepwise regression analysis	Logistic regression model	Hosmer– Lemeshow test	Internal validation	APACHEII, SOFA, Middle cerebral artery PI, Arterial blood lactate, ALT, rScO2, Albumin	B,0.831 (0.773–0.889)	Nomogram model
Zhao, Qing (2023)	Direct exclusion	–	Logistic regression model	Calibration curve analysis	Internal validation	Age, SOFA, Na+, HR, T	A, 0.802 B, 0.809	Nomogram model
Xiao, Lu (2022)	Multiple interpolation	–	GBDT XGBoost Light-GBM SVM DT RF	–	Internal validation	The top 5 factors ranked by importance are as follows: mechanical ventilation, duration of mechanical ventilation, serum phosphorus level, SOFA, vasopressor	A, 0.883 (0.847–0.896) 0.902 (0.883–0.919) 0.879 (0.864–0.887) 0.832 (0.824–0.857) 0.849 (0.838–0.868) 0.886 (0.871–0.890) B, 0.872 (0.859–0.885) 0.884 (0.865–0.898) 0.877 (0.869–0.888) 0.818 (0.808–0.839) 0.847 (0.839–0.855) 0.874 (0.868–0.881)	Script
Zhang, Li (2024)	–	–	Logistic regression model	–	–	APACHE II, SOFA, CCI, lung infection, Hb	A,0.975 (0.931–1)	–
Jin, Jun (2024)	Multiple interpolation	LASSO regression	Multivariable Logistic regression model	Hosmer– Lemeshow test Calibration curve analysis	Internal validation	Gender, Age, BMI, MAP, T, Platelet count, Na+, Midazolam use, SOFA	A,0.751 (0.734–0.768) B,0.766 (0.74–0.793)	Nomogram model
Zhao, Li na (2021)	Mean value interpolation	LASSO regression	Multivariable Logistic regression model	Calibration curve analysis	Internal validation	Age, Carbapenem, antibiotics, Quinolone antibiotics, qSOFA, midazolam, H antagonists, Steroids, Phenylephrine, hydrochloride, Heparin sodium injection	A,0.743 (0.72–0.766) B,0.762 (0.716–0.807)	Nomogram model
Liu, Xiaoyu (2021)	Direct exclusion	–	Multivariable Logistic regression model	–	–	APACHE II, CD86MFI, Albumin	A,0.894 (0.817–0.97)	Formulas
Mei, Jiang jun (2024)	–	–	Multivariable Logistic regression model	Hosmer– Lemeshow test Calibration curve analysis	Internal validation	CCT, PI, S100β	B,0.924 (0.833–0.975)	Nomogram model
Ge, Cheng long (2022)	Single interpolation	–	Logistic regression model SVM DT RF GBM MLP XGBoost LGBM	Calibration curve analysis	Internal validation	GCS, Glucose, Age, Mean arterial pressure, Mean heart rate, Hemoglobin, Length of stay in hospital, Length of stay in ICU, Platelet, WBC, PO2, Weight, Liver disease, PH, Resprate mean	A,0.74 0.72 0.71 0.75 0.77 0.76 0.91 B,0.74 0.71 0.72 0.86 0.87 0.69 0.85 0.87	Script

**Notes.**

SVC Support vector machine DT Decision Tree RF Random Forest GBM Gradients Boosting Machine MLP Multiple Layer Perception GBDT Gradient Boosting Decision Tree SVM Support Vector Machine S100β S100 Calcium-Binding Proteinβ ALT Alanine Aminotransferase HR Heart Rate T Temperature LOS ICU Stay Time CCI Charlson Comorbidity Index Hb Hemoglobin. BMI Body Mass Index MAP Mean Arterial Pressure qSOFA Quick Sepsis Related Organ Failure Assessment CD86MFI CD86 Mean Fluorescence Intensity WBC White Blood Cell

A, development cohort. B, validation cohort.

Note. Wang, Zhao & Chao, 2023; Zhao et al., 2023; Zhou et al., 2023; Lu et al., 2022; Zhang et al., 2024; Jin et al., 2024; Zhao et al., 2021; Liu, 2021; Mei et al., 2024; Ge et al., 2022.

### Model validation

Among the included studies, eight conducted internal validation using different approaches. Three studies employed the bootstrap method, five used random split validation, and two utilised cross-validation. None of the models underwent external validation. Reported AUC values ranged from 0.743 to 0.975, with 9 models exceeding 0.75, indicating strong discriminatory performance. Seven studies assessed model calibration, primarily using calibration curves. Among these, four also reported Hosmer–Lemeshow test *p*-values > 0.05 (range: 0.126–0.944), indicating good agreement between predicted and observed risks. Four studies further evaluated the clinical utility of the models *via* Decision-Curve Analysis (DCA), demonstrating that the models provided a positive net benefit across clinically relevant probability thresholds.

### Results of quality assessment

Table 3 summarises the quality level, risk of bias, and applicability of the included studies. All studies were assessed as having a high risk of bias, mainly due to issues identified in the participant and analysis domains, as detailed below.

**Table 3:** PROBAST results of the included studies.

Author (year)	Study	ROB				Applicability			Overall	
		Participants	Predictors	Outcome	Analysis	Participants	Predictors	Outcome	ROB	Applicability
Wang, Ziwen (2023)	B	–	+	–	–	+	+	+	–	+
Zhou, Hang xiang (2023)	B	–	+	+	–	+	+	+	–	+
Zhao, Qing (2023)	B	–	+	–	–	+	+	+	–	+
Xiao, Lu (2022)	B	–	+	–	–	+	+	+	–	+
Zhang, Li (2024)	B	–	+	+	–	+	+	+	–	+
Jin, Jun (2024)	B	–	+	–	+	+	+	+	–	+
Zhao, Lina (2021)	B	–	+	–	+	+	+	+	–	+
Liu, Xiaoyu (2021)	B	+	+	+	–	+	+	+	–	+
Mei, Jiangjun (2024)	B	+	–	–	–	+	+	+	–	+
Ge, Chenglong (2022)	B	–	+	–	–	+	+	+	–	+

**Notes.**

PROBAST Prediction model Risk Of Bias Assessment Tool B moderate ROB risk of bias

+indicates low ROB/low concern regarding applicability.

-indicates high ROB/high concern regarding application.

Note. Wang, Zhao & Chao, 2023; Zhao et al., 2023; Zhou et al., 2023; Lu et al., 2022; Zhang et al., 2024; Jin et al., 2024; Zhao et al., 2021; Liu, 2021; Mei et al., 2024; Ge et al., 2022.

In the participant domain, the majority of studies were at high risk of bias, mainly attributable to their retrospective design (Ge et al., 2022; Jin et al., 2024; Lu et al., 2022; Wang, Zhao & Chao, 2023; Zhang et al., 2024; Zhao et al., 2021; Zhao et al., 2023; Zhou et al., 2023). In the predictor domain, one study was deemed high risk due to potential information bias in its predictors (Mei et al., 2024). In the outcome domain, seven studies were at high risk of not adequately separating predictor variables from the outcome definition (Ge et al., 2022; Jin et al., 2024; Lu et al., 2022; Mei et al., 2024; Wang, Zhao & Chao, 2023; Zhao et al., 2021; Zhao et al., 2023).

The analysis domain presented the most widespread concerns, with all studies exhibiting a high risk of bias. Key methodological shortcomings included an insufficient sample size relative to the number of predictor variables, insufficient reporting of missing-data handling, omission of calibration performance measures, lack of model validation, and reliance on univariate screening.

Regarding applicability, all models were rated low across domains, indicating strong relevance to the target clinical scenario. For instance, the machine learning model developed by Lu et al. demonstrated not only high performance but also enhanced interpretability through SHAP analysis and clinical review, underscoring its potential utility.

### Meta-analysis

**Figure 2:**
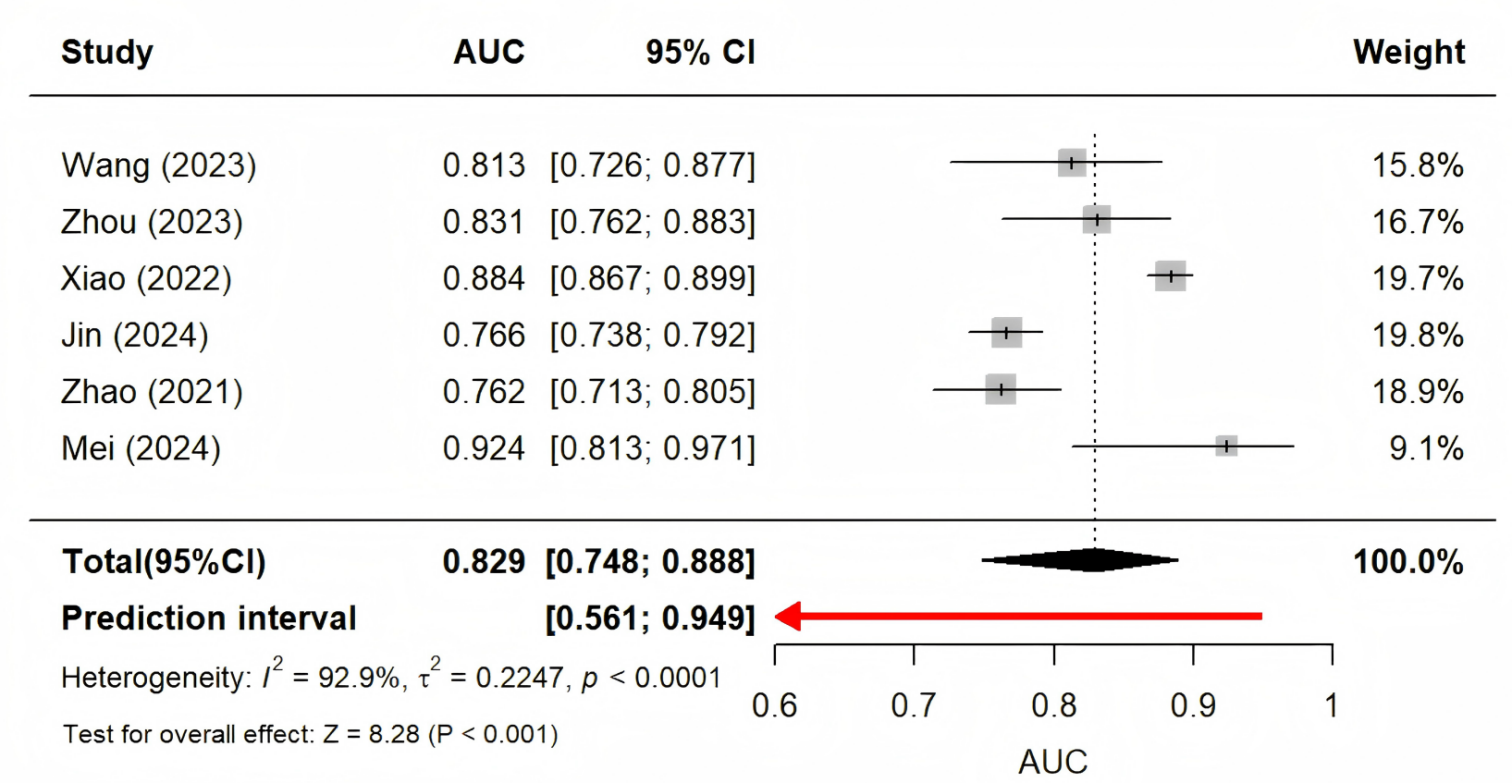
AUC’s forest diagram.

**Figure 3:**
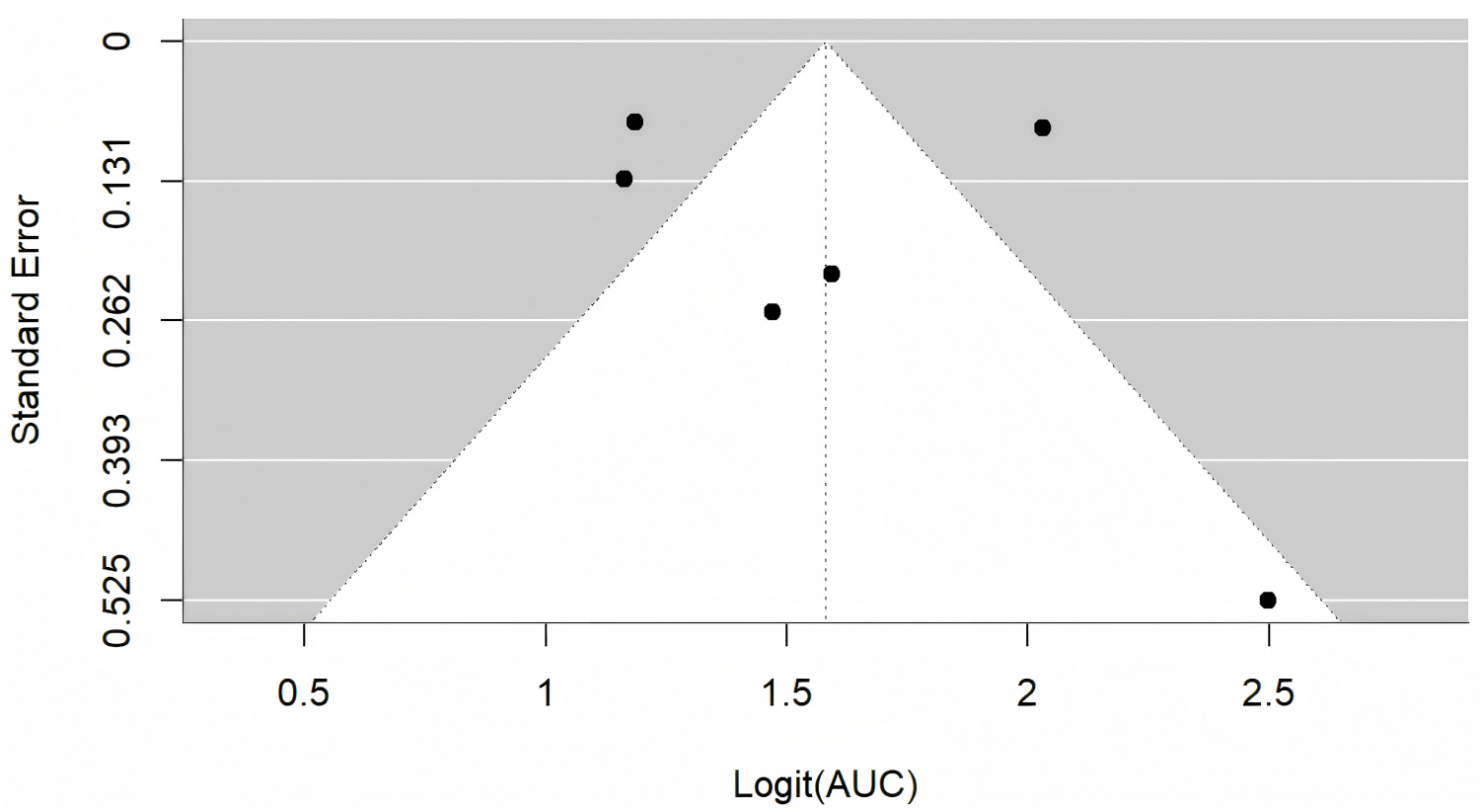
Funnel diagram.

Four studies were excluded from the meta-analysis due to insufficient reporting of model validation details. Two studies involved multiple models, and all methods were based on the same samples; therefore, only the best-performing XGBoost and Light Gradient Boosting Machine (LGBM) models were included. Consequently, six studies that provided adequate validation data were synthesized (Jin et al., 2024; Lu et al., 2022; Mei et al., 2024; Wang, Zhao & Chao, 2023; Zhao et al., 2021; Zhou et al., 2023). Using a random-effects model, the combined AUC was (95% CI [0.77–0.89]) (Fig. 2). The *I*^2^ value was 93.3% (*p* < 0.001), indicating a high degree of heterogeneity between the studies. Sensitivity analysis, performed by sequentially excluding each study, showed minimal change in the overall results, suggesting the meta-analysis was robust. The Egger’s test yielded a value of 0.2699 (*p* > 0.05), indicating a low likelihood of publication bias (Fig. 3).

## Discussion

The number of SAE prediction models has been steadily increasing; however, their clinical practice, quality, and applicability are still unknown. This study is the first to systematically evaluate the methodological quality of SAE prediction models and provide evidence-based recommendations for model optimization.

During the process of constructing the SAE model, several noteworthy strengths are worth learning from. For example, Jin et al. (2024) included a large sample size their retrospective design increased the potential for bias. However, their study excelled in analysis by using multiple imputation for missing data, an approach often neglected in similar studies. Mei et al. (2024) employed Least Absolute Shrinkage and Selection Operator (LASSO) regression for variable selection, which offers notable advantages in terms of predictive accuracy and model interpretability (Emmert-Streib & Dehmer, 2019). In contrast, two studies performed direct deletion of missing data; however, this approach has been shown to perform poorly in terms of calibration and predictive accuracy. Future studies could utilize optimal performance methods, such as multiple imputation for missing values (Deforth, Heinze & Held, 2024). Notably, Lu et al. (2022) and Ge et al. (2022) applied machine learning (ML) methods during model development. It has been shown that machine learning methods tend to achieve higher accuracy than traditional logistic regression (Churpek et al., 2016), owing to their superior ability to capture nonlinear relationships, handle high-dimensional data, and automate variable selection. However, one of the drawbacks of machine learning models is their lack of interpretability, and many ML scientists agree that “black boxes” are one of the main barriers to the adoption of ML in medicine (Vellido, 2020). Therefore, the development of interpretable ML models is an urgent need nowadays. Lu et al. (2022) employed the SHAP method to interpret the outputs of the constructed ML models and invited clinicians to rate them, thereby further enhancing the models’ interpretability. This combined strategy—SHAP-based interpretation coupled with expert evaluation—represents a promising framework for improving transparency in ML-driven prediction models.

The existing predictive models reported in this review also have important clinical implications. The high-frequency predictors shown are informative for future nursing practice and clinical diagnostic studies. The SOFA score is a commonly used method for assessing the degree of organ failure in patients with sepsis; although SOFA does not directly diagnose SAE, the severity of sepsis as assessed by the SOFA has been associated with the presence or absence of SAE, higher SOFA scores have been associated with SAE occurrence (Leventogiannis et al., 2022). The study by Zhao et al. (2021) included both SOFA scores and qSOFA scores; ultimately, qSOFA was used to construct the model, whereas SOFA did not emerge as a significant predictor—an observation that warrants further investigation to inform model development. The SOFA score, as a robust and readily available clinical metric, shows consistent predictive value for SAE and should be considered a cornerstone variable in future risk stratification tools. Age is a well-established susceptibility factor for SAE, and the incidence of SAE gradually increases after the aged ≥50 years, which is associated with a decline in brain reserve, a physiological decline in the function of most organs, and concomitant complications (Schütze et al., 2023). Therefore, healthcare professionals should be alert to septic patients aged 50 years and older.

Sodium (Na^+^) is critical for plasma osmolality regulation. Hypernatremia increases plasma osmolality and can drive intracellular water shifts in neurons, precipitating neurological dysfunction; Yang et al. (2020) reported a significant association between hypernatremia and SAE (Yang et al., 2020). APACHE II score and body temperature were used as predictors in three studies. Fever commonly reflects systemic infection and may disrupt cerebral metabolism; prolonged hyperthermia can exacerbate neuronal injury and compromise the blood–brain barrier (Sun et al., 2013). Higher APACHE II scores reflect greater overall disease severity and have been linked to increased mortality and SAE risk (Liu, 2021; Sonneville et al., 2017). Future model development should integrate these key predictors and further examine their mechanistic roles and interactions to improve predictive performance and clinical applicability.

The clinical significance of these individual predictors ultimately manifests in how they are selected and integrated into multivariable models. An examination of the six predictive models included in this meta-analysis (Jin et al., 2024; Lu et al., 2022; Mei et al., 2024; Wang, Zhao & Chao, 2023; Zhao et al., 2021; Zhou et al., 2023) revealed substantial discrepancies in their construction strategies. Specific models integrated organ dysfunction scores (SOFA/APACHE II), routine laboratory parameters, and demographic data to synthesise commonly available clinical information. By contrast, other models focused on pathophysiological mechanisms, emphasising cerebral hemodynamics or immune biomarkers. Notably, Zhao et al. (2021) incorporated treatment-related variables, highlighting the potential impact of clinical interventions on the risk of sepsis-associated encephalopathy (SAE). The diversity in predictor selection not only corroborates the multifactorial nature of SAE but also partially accounts for the significant statistical heterogeneity observed in this meta-analysis. Simultaneously, it underscores the current lack of a consensus regarding the core variables for SAE prediction.

This review also identified several significant issues with existing SAE models. We included 10 studies and conducted a meta-analysis of six predictive models (Jin et al., 2024; Lu et al., 2022; Mei et al., 2024; Wang, Zhao & Chao, 2023; Zhao et al., 2021; Zhou et al., 2023). The ten included studies reported AUC values ranging from 0.786 to 0.988. However, all studies were deemed high-risk according to the PROBAST checklist, limiting the practical applicability of predictive models. The pooled AUC of the six validated models was 0.83 (95% CI [0.77–0.89]), demonstrating moderate predictive performance but substantial heterogeneity. Such heterogeneity likely arises from differences in study design (predominantly retrospective), patient characteristics, and data quality. Addressing such heterogeneity and improving model generalizability requires systematic multicenter external validation.

All included studies were authored by Chinese researchers, potentially reflecting China’s growing concern regarding SAE (Mazeraud et al., 2020). This heightened concern arises from the high prevalence and clinical burden of sepsis in the Chinese population, where SAE frequently occurs as one of its most severe complications. The reported incidence of sepsis in China remains persistently high, mainly due to its large population, aging demographics, and high rates of comorbidities (Liao et al., 2016; Weng et al., 2023). Elderly patients, in particular, are more vulnerable to SAE due to diminished physiological reserves and pre-existing conditions (Gu et al., 2025). China’s issues with antibiotic resistance and uneven distribution of medical resources further contribute to the high incidence of sepsis (Luo et al., 2024; Weng et al., 2023).

Consequently, Chinese researchers have been actively developing early diagnostic models to mitigate the risk of misdiagnosis and improve clinical outcomes. Furthermore, the availability of international open-access databases, such as Medical Information Mart for Intensive Care (MIMIC), has facilitated the development of SAE prediction models. However, most of these models remain reliant on retrospective, single-centre datasets and lack external validation across diverse populations and healthcare systems, which limits their generalizability and clinical applicability.

In summary, the two major issues identified in this review—high heterogeneity and high risk of bias—suggest that future studies must undergo multicenter external validation and adopt rigorous research designs. External validation is essential to determine whether the observed heterogeneity arises from model overfitting or from clinical differences in patient populations and healthcare settings. Validating models across centres in different regions and healthcare environments allows direct assessment of their generalisation capabilities in patient populations and settings distinct from the development cohort. Moreover, validation across diverse clinical settings can assess the robustness of predictive performance and thereby determine broad clinical applicability (Debray et al., 2015). Importantly, external validation also identifies meaningful variations across environments, driving the development of more universal models or guiding adjustments tailored to specific contexts.

High bias risk primarily stems from suboptimal study designs, underscoring the need for rigorous research methodologies in future work. Currently, most studies are retrospective, which may introduce bias in participant selection and data quality. Future research should therefore prioritize prospective study designs, which can mitigate bias and ensure more comprehensive data collection. In addition, *a priori* sample size calculations should be performed to ensure reliability (Talari & Goyal, 2020). To maintain data integrity, advanced techniques such as multiple imputation should be employed to handle missing values appropriately. Furthermore, reliance on univariate analysis for variable selection may omit important predictors or overemphasize less relevant ones (Chang & Chen, 2025). Techniques such as the least absolute shrinkage and selection operator regression should be employed for more reliable variable selection, particularly in high-dimensional datasets.

### Limitations

This study has several limitations. First, it only included literature in Chinese and English, which may have introduced language bias. Due to significant heterogeneity among the studies, quantitative analysis was not performed. Furthermore, most of the included studies were retrospective, single-centre investigations lacking external validation, which may limit the generalizability and clinical applicability of the models. Adjustments may be necessary when applying these models in different regions.

## Conclusions

This systematic review evaluated 10 studies. For the six models subjected to validation, the pooled AUC was 0.83 (95% CI [0.77–0.89]), reflecting moderate discriminatory capability. However, according to PROBAST and GRADE assessments, all studies were classified as having a high risk of bias and low methodological quality. To translate this predictive potential into clinical utility, researchers should strengthen study design, perform multicenter external validation, and adhere to the PROBAST framework to enhance the credibility and clinical utility of future models.

## Supplemental Information

10.7717/peerj.20770/supp-1 Supplemental Information 1 PRISMA checklist

10.7717/peerj.20770/supp-2 Supplemental Information 2 Pubmed history

## Additional Information and Declarations

### Data Availability

The following information was supplied regarding data availability: This is a systematic review/meta-analysis.
